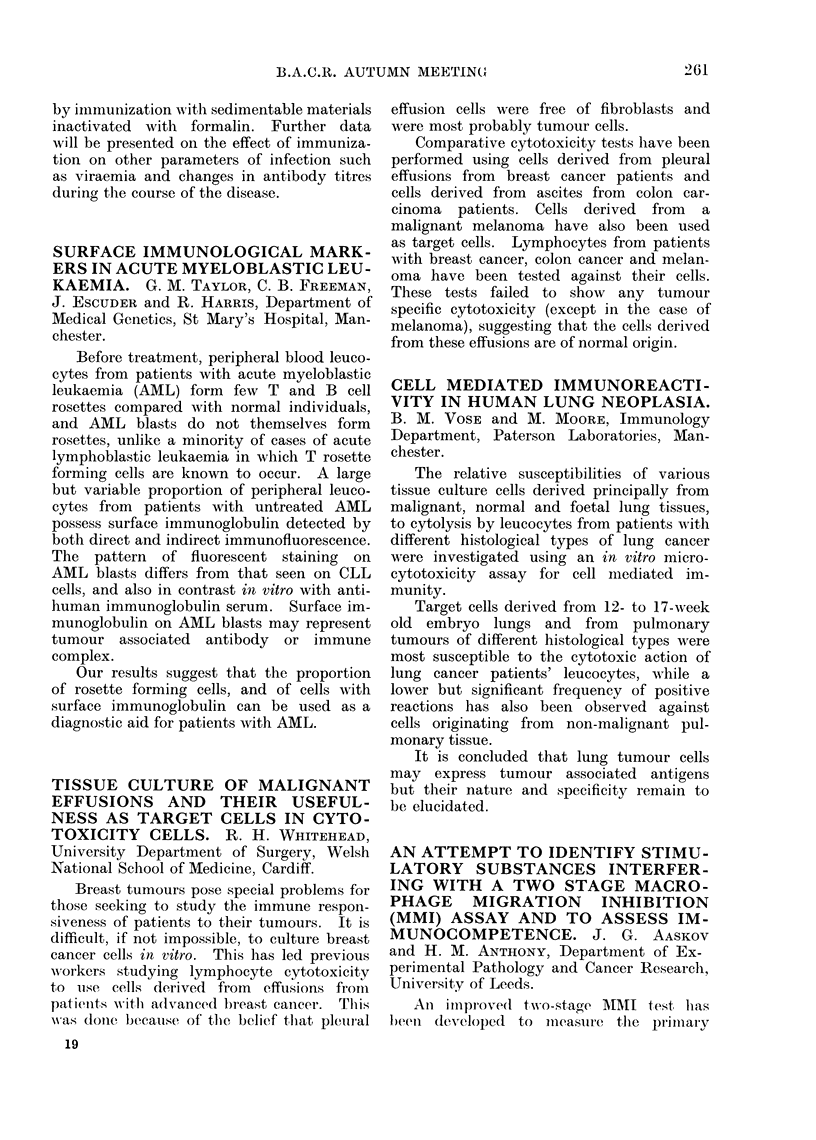# Proceedings: Surface immunological markers in acute myeloblastic leukaemia.

**DOI:** 10.1038/bjc.1975.39

**Published:** 1975-02

**Authors:** G. M. Taylor, C. B. Freeman, J. Escuder, R. Harris


**SURFACE IMMUNOLOGICAL MARKERS IN ACUTE MYELOBLASTIC LEUKAEMIA.** G. M. TAYLOR, C. B. FREEMAN, J. ESCUDER and R. HARRIS, Department of Medical Genetics, St Mary's Hospital, Manchester.

Before treatment, peripheral blood leucocytes from patients with acute myeloblastic leukaemia (AML) form few T and B cell rosettes compared with normal individuals, and AML blasts do not themselves form rosettes, unlike a minority of cases of acute lymphoblastic leukaemia in which T rosette forming cells are known to occur. A large but variable proportion of peripheral leucocytes from patients with untreated AML possess surface immunoglobulin detected by both direct and indirect immunofluorescence. The pattern of fluorescent staining on AML blasts differs from that seen on CLL cells, and also in contrast *in vitro* with anti-human immunoglobulin serum. Surface immunoglobulin on AML blasts may represent tumour associated antibody or immune complex.

Our results suggest that the proportion of rosette forming cells, and of cells with surface immunoglobulin can be used as a diagnostic aid for patients with AML.